# Pericentrin Is Related to Abnormal β-Cell Insulin Secretion through F-Actin Regulation in Mice

**DOI:** 10.1371/journal.pone.0130458

**Published:** 2015-06-17

**Authors:** Yuan Zu, Yanping Gong, Lijuan Wan, Yang Lv, Shaoyuan Cui, Xinye Jin, Chunlin Li, Xiangmei Chen

**Affiliations:** 1 Department of Geriatric Endocrinology, Chinese People's Liberation Army General Hospital, Beijing, 100853, China; 2 Department of Nephrology, Chinese People's Liberation Army General Hospital, Chinese People's Liberation Army Institute of Nephrology, State Key Laboratory of Kidney Diseases, National Clinical Research Center for Kidney Diseases, Beijing, 100853, China; 3 Department of Internal, Chinese People's Liberation Army General Hospital, Beijing, 100853, China; National Center for Scientific Research Demokritos, GREECE

## Abstract

The aim of this study was to investigate the regulating effect of pericentrin (PCNT) on insulin secretion in the development of insulin resistance and to determine the underlying mechanism. PCNT expression was studied in different tissues of C57/B6 mice by reverse transcriptase-PCR and immunofluorescence. PCNT was highly expressed in organs involved in the regulation of metabolism, while cytoplasmic expression was only enriched in islet cells. PCNT expression was significantly lower in the central regions of insulin resistance (IR) mouse islets than in those of control mouse islets. PCNT expression was further studied in mouse MIN6 cells exposed to glucose stimulation, small interfering RNA (siRNA) against PCNT, and an ERK inhibitor (PD98095). The results revealed that PCNT expression in glucose-stimulated MIN6 cells reduced linearly with cytoplasmic insulin levels. MIN6 cells transfected with PCNT siRNA showed significantly decreased intracellular insulin and F-actin expression. The change in F-actin expression in MIN6 cells during PCNT siRNA interference showed a linear relationship with PCNT expression at different time points. The ERK inhibitor affected PCNT expression and F-actin expression linearly. The abnormal insulin secretion observed both *in vivo* and *in vitro* was associated with decreased PCNT expression, and F-actin was found to be the target of PCNT regulation.

## Introduction

Diabetes has become a disease of worldwide prevalence. In 2013, 382 million people had developed diabetes, with type 2 diabetes mellitus (T2DM) comprising 90% of all cases, which is equivalent to 8.3% of the entire global adult population [1,2]. Diabetes results in approximately 1.5 million deaths annually [2] and the percentage of adults with diabetes is increasing. It is anticipated that the number of annual deaths resulting from diabetes may nearly double by 2030 [3]. Serious research efforts have been devoted to overcoming diabetes, although progress has been hindered because of glucose fluctuates, causing variable cases of hyperglycemia and hypoglycemia [4]. Thus, investigations into regulatory mechanisms underlying insulin homeostasis have been the subject of great interest in recent years.

There are two phases of pancreatic beta cells’ insulin secretion. During the first phase, insulin granules are released from the established pool near the plasma membrane (PM). Whereas in the second-phase, it was proposed that insulin granules came from the newly synthesized pools deep in the cells, and were transferred continuously from storage pools towards the PM [5–8].

Pericentrin (PCNT), a component of the pericentriolar material, is a highly conserved coiled coil protein [9–13]. PCNT localizes to centrosomes and serves as a multifunctional scaffolding protein for various proteins and protein complexes [14]. Recently, loss-of-function studies of human PCNT have established a causal link between PCNT mutations and the early onset of T2DM in microcephalic osteodysplastic primordial dwarfism type II (MOPD II) patients [15,16]. The mechanism linking pericentrin mutations with dysregulation of glucose homeostasis, however, is still unknown.

This study was designed to determine whether changes in PCNT expression occur in high-fat-induced insulin resistant (IR) mice *in vivo* and to explore the *in vitro* mechanism whereby PCNT regulates insulin secretion, using a mouse pancreatic cell line (MIN6).

## Materials and Methods

### Animals

Twenty-four male specific-pathogen-free (SPF) C57BL/6 mice, aged 6–8 weeks and weighing 22 ± 2 g (mean ± SD), were purchased from the Institute of Laboratory Animal Sciences at the Chinese Academy of Medical Sciences (Beijing, China). Mice were housed in individual cages, under conditions of constant temperature (23°C ± 1°C) and humidity (50% ± 5%) in a standard 12-h light/12-h dark cycle with free access to food and water. Food was supplied every day after their cages were cleaned and before the dark cycle began. Animal care and experimentation were conducted in accordance with the National Institute of Health Guide for the Care and Use of Laboratory Animals (http://oacu.od.nih.gov/regs/guide/guide4.htm), and consent was obtained from the Ethics Committee of the Chinese People’s Liberation Army General Hospital in Beijing, China. All efforts were made to minimize suffering.

### Animal treatment and sample collection

Mice were randomly divided into 2 groups (n = 12 per group) using computer-generated random numbers. The normal (N) group was fed a standard diet (329.0 kcal/100 g of food; 12.3% of calories from fat, 24.4% from protein, and 63.3% from carbohydrate), and the IR group was fed a high-fat diet (414.0 kcal/100 g of food; 38.0% of calories from fat, 12.0% from protein, and 50.0% from carbohydrate). Mice were maintained on these diets for 12 weeks and were then sacrificed by CO_2_ after glucose tests listed as below.

Glucose tolerance tests (GTTs), including the intraperitoneal glucose tolerance test (IPGTT) and the oral glucose tolerance test (OGTT) were performed on all animals, as described below, every 2 weeks, commencing after 4 weeks of feeding. Blood samples (~ 1 ml) for insulin testing were obtained by eye enucleation without the use of an anticoagulant, and serum samples were prepared by centrifuging at 2000 rpm (4°C for 15 min) and stored at −20°C for subsequent measurements of insulin levels. Six mice from each group were randomly selected for IPGTT and OGTT tests.

Tissue samples (adipose, spleen, kidney, liver, skeletal muscle, pancreas, intestinal, diaphragm, and testis) were obtained from all mice sacrificed in week 12. All tissue samples were snap frozen in liquid nitrogen and stored at −80°C for subsequent reverse transcription PCR (RT-PCR), as described below. Pancreas samples were also investigated by gene chips using ArrayCompass System (Affymetrix, Santa Clara, USA) of the CapitalBio Corporation (Beijing, China). Tissues used for immunohistochemistry (IHC) studies were fixed in 10% neutral-buffered formalin (NBF) for at least 3 days before being embedded in paraffin. Tissues for immunofluorescence studies were submerged in optimal cutting temperature (OCT) compound (Biogen Idec, Cambridge, MA, USA), cooled in liquid nitrogen, and stored at -80°C.

### Glucose tolerance tests

Glucose tolerance tests were performed on all animals. Mice were fasted for 10–12 h overnight. The IPGTT was used to evaluate the first phase of insulin secretion and the OGTT was used to test the second phase of insulin secretion. Three mice from each of the N and IR groups were sacrificed at 0 min to determine baseline glucose and insulin levels. Approximately 2 g/kg body weight glucose was administered intraperitoneally or intragastrically to 3 mice from the N and IR groups. Glucose levels were tested from the tail vein of each mouse at 0, 2, 4, 6, 8, 10, and 15 min in IPGTT. Blood samples were tested at 0, 30, 60, and 120 min in OGTT. Mice were then sacrificed for subsequent insulin level testing.

Blood glucose levels were tested using a standard glucometer (Accu-Chek, Roche, Mannheim, Germany). Serum insulin levels were measured by radioimmunoassay (RIA), using the Sensitive Rat Insulin RIA Kit (SRI-13K; Millipore, Billerica, MA) according to the manufacturer’s instructions. Homeostasis model assessment for insulin resistance (HOMA-IR) values were determined from results of the fasting blood glucose (FBG) and fasting insulin (FINS) tests, using the equation HOMA-IR = (FBG × FINS)/22.5.

### Quantitative RT-PCR (Q-RT-PCR)

Total RNA from islets was extracted using the RNeasy Micro Kit (Qiagen, Madrid, Taiwan) and Q-RT-PCRs were performed according to manuscript. Briefly, 2 μg RNA was used for first-strand cDNA synthesis using Superscript II (Invitrogen S.A.) in a total volume of 20 μl. Three microliters of diluted cDNA sample was used as a template for Q-RT-PCR, and detection was achieved with SYBR green (Roche Farma S.A., Madrid, ES). Q-RT-PCR data were normalized using the ΔΔCt method (2^-ΔΔCt^), which gives relative expression levels for transcripts evaluated in different tissues. Primer sequences listed in Table 1 were designed using Primer 3 Software (http://frodo.wi.mit.edu/primer3/). Glyceraldehyde 3-phosphate dehydrogenase (GAPDH) was used as the internal control for determining relative target gene expression levels via the ΔΔCt method.

**Table 1:** Sequences of primers used in this study.

Gene		Primers (5′–3′)
Pericentrin	Forward	CGGGCAAGGAAAGATCAACTTCG
	Reverse	TGAGTAGAATCTGGCGGCAACC
ERK	Forward	GACCTCATGGAGACGGACCTTTAC
	Reverse	TCACAAGTGGTGTTCAGCAGGAG
F-actin	Forward	GGTAGAGTTGGCTTTATGGGACAC
	Reverse	CAGGATGATGGGCACATTTGGAC
Cyclin D2	Forward	AGTCCCGACTCCTAAGACCC
	Reverse	TGGGGCTTCACAGAGTTGTC
P21	Forward	GGTGATGTCCGACCTGTTCCG
	Reverse	CCAGACGAAGTTGCCCTCCAG
CDK4	Forward	GCAGTCAGTGGTGCCAGAGATG
	Reverse	TGCGTCGCTTTCCTCCTTGTG

### Western blots

Tissue samples (approximately 0.5 cm^3^ from each mouse) were washed in PBS and lysed by sonication in 100 μl of ice-cold lysis buffer containing Tris-HCl (pH 7.5), 150 mM NaCl, 1 mM EDTA, 2 mM DTT, 2 mM PMSF, and 1% Triton X-100. The lysates were centrifuged at 12,000 × *g* for 30 min at 4°C. Protein concentrations for each sample were determined using the BCA Protein Assay Kit (Thermo Fisher Scientific, Rockford, IL). Equal amounts of protein were resolved on SDS-PAGE gels, transferred to nitrocellulose membranes (Amersham, Little Chalfont, UK), and membranes were blocked in 5% skim milk power in PBS/Tween for 1 h at room temperature. The membranes were then incubated overnight at 4°C in PBS/Tween with 2% BSA containing the following dilutions of primary antibodies from Abcam (Cambridge, MA): anti-β-actin (1: 1000), anti-Cyclin D2 (1: 1000), and anti-CDK (1: 1000). The membranes were washed in PBS/Tween 3 times for 6 min/wash, Membranes were then incubated for 1 h at room temperature with 1: 5000 dilutions of horseradish peroxidase (HRP)-conjugated rabbit anti-mouse IgG secondary antibodies (Beyotime, Beijing, China). Next, membranes were washed in PBS/Tween 3 times for 6 min/wash, and the bands of interest were visualized by enhanced chemiluminescence (eBioscience, San Diego, CA).

### Tissue immunohistochemistry

Mouse tissues were fixed in NBF for at least 3 days, embedded in paraffin, and sectioned at 7 μm. Tissue sections were deparaffinized and rehydrated using xylene and methanol. Endogenous peroxidase activity in tissue sections was inhibited by incubation in 3% H_2_O_2_ for 10 min. Subsequently, tissue sections were incubated in potassium citrate solution as antigen retrieval buffer in a microwaveable vessel. The vessels containing the slides were microwaved/boiled for 20 min and cooled at room temperature in PBS for 10 min. Tissue samples were blocked in PBS with 3% BSA for 1 h. Primary antibodies against insulin, glucagon, somatostatin (Santa Cruz Biotechnology, Santa Cruz, CA) or PCNT (BD Bioscience, San Jose, CA) were diluted 1:200, 1:200, 1:50, or 1:500 in PBS, and incubated with tissue samples overnight at 4°C. Tissues were washed 3 times in PBS and incubated with appropriate anti-rabbit or anti-mouse biotinylated secondary antibodies (Beyotime, Beijing, China, 1: 300 dilution in PBS) for 1 h at room temperature. Staining was achieved by incubating slides with streptavidin-conjugated HRP for 40 min and subsequently with DAB for 5 min. Slides were analyzed using a U-RFL-T microscope (Olympus; Tokyo, Japan).

### MIN6 cell culture

MIN6 cells were obtained from Dr. Xinyu Miao (Department of Geriatric Endocrinology, Chinese People's Liberation Army General Hospital, Beijing, China) [17]. MIN6 cells were maintained in Dulbecco’s modified Eagle medium (DMEM; 25 mM glucose; Gibco), supplemented with 15% FCS, penicillin/streptomycin (100 units/ml, 0.1 mg/ml), 2 mM L-glutamine, and 0.05 mM 2-mercaptoethanol. Cells were incubated at 37°C in a 5% CO_2_-humidified incubator.

To study relationships between PCNT and insulin secretion, as well as the effect of F-actin, glucose stimulation was performed as follows: MIN6 cells were planted in 6-well chamber plates at about 50% confluent. Cells were then cultured in DMEM (Gibco) with 5 mM, 15 mM, or 25 mM glucose for 48 h. MIN6 cells were double-stained with antibodies against PCNT and insulin, or PCNT and F-actin. MIN6 cells were transfected with small-interfering RNAs (siRNAs) against mRNAs of PCNT or a scrambled control for 48 h, after which they were also double-stained. Duplicate wells of MIN6 cells were also incubated with 50 μM PD98095 (an ERK inhibitor) and stained with appropriate antibodies to study the effects of ERK inhibition on PCNT and F-actin expression.

### RNA interference (RNAi)-mediated PCNT silencing

MIN6 cells were transfected with PCNT siRNA or scrambled siRNA using Lipofectamine RNAiMAX Reagent (Invitrogen, Carlsbad, CA), according to the manufacturer’s instructions. Briefly, cells were cultured in 6-well plates until they reached 60–80% confluence. Nine microliters of Lipofectamine RNAiMAX Reagent was diluted in 150 μl Opti-MEM Medium, and 3 μl PCNT siRNA and scrambled siRNAs were diluted in separate 150 μl volumes of Opti-MEM Medium. Each diluted siRNA solution was mixed with separate solutions of diluted Lipofectamine RNAiMAX Reagent and incubated for 5 min at room temperature. Subsequently, the siRNA-lipid complexes were added to cells and cells were incubated at 37°C. MIN6 cells were harvested at 0, 24, and 48 h post-transfection and used for double staining experiments against PCNT and insulin, or PCNT and F-actin, as described below.

### Double immunofluorescence staining

Cells were grown in glass-bottom culture dishes at 37°C and 5% CO_2_ for the indicated times. Cells were washed in PBS, fixed/permeabilized on ice for 30 min in fixation solution (2% paraformaldehyde in PBS), washed 4 times in PBS, and blocked for 15 min with 1% BSA in PBS. Fixed cells were incubated at 4°C overnight with primary antibodies against insulin (Santa Cruz Biotechnology, CA), PCNT (Abcam, Cambridge, MA), or F-actin (Santa Cruz Biotechnology, CA) that were diluted 1: 200, 1: 500, or 1: 1000 in PBS with 1% BSA. Cells were then incubated for 1 h in PBS containing 1% BSA and appropriate secondary antibodies (1: 500 dilution, P0196, FITC-conjugated anti-mouse IgG; P0193, Cy3-conjugated anti-mouse IgG; P0186, FITC-conjugated anti-rabbit IgG; and P0183, Cy3-conjugated anti-rabbit IgG, Beyotime, Beijing, China). Nuclei were stained with DAPI (Beyotime, Beijing, China). The slides were analyzed using a microscope (Olympus U-RFL-T, Tokyo, Japan). Confocal imaging was performed with a confocal microscope (Radiance 2000, BioRad, California, USA) using a 60 × CFI plan Apo objective and a filter optimized for mCherry fluorescence.

Fresh tissues were submerged in OCT compound (Biogen Idec, Cambridge, MA), cooled in liquid nitrogen, and stored at -80°C until further use. Cryostat sections (4–8 μm thick) were mounted on Superfrost Slides. Slides were warmed to room temperature for 30 min before IF staining was initiated, as described above. Slides were analyzed using an Olympus U-RFL-T Microscope.

### Statistical analysis

All data analyses were performed using SPSS 17.0 software (SPSS, Inc., Chicago, IL). Data are presented as means ± SD. Comparisons were made using unpaired Student’s t tests and one-way ANOVA, as appropriate. *P*-values less than 0.05 were considered statistically significant.

## Results

### High cytoplasmic PCNT expression in islet cells

**Fig 1:**
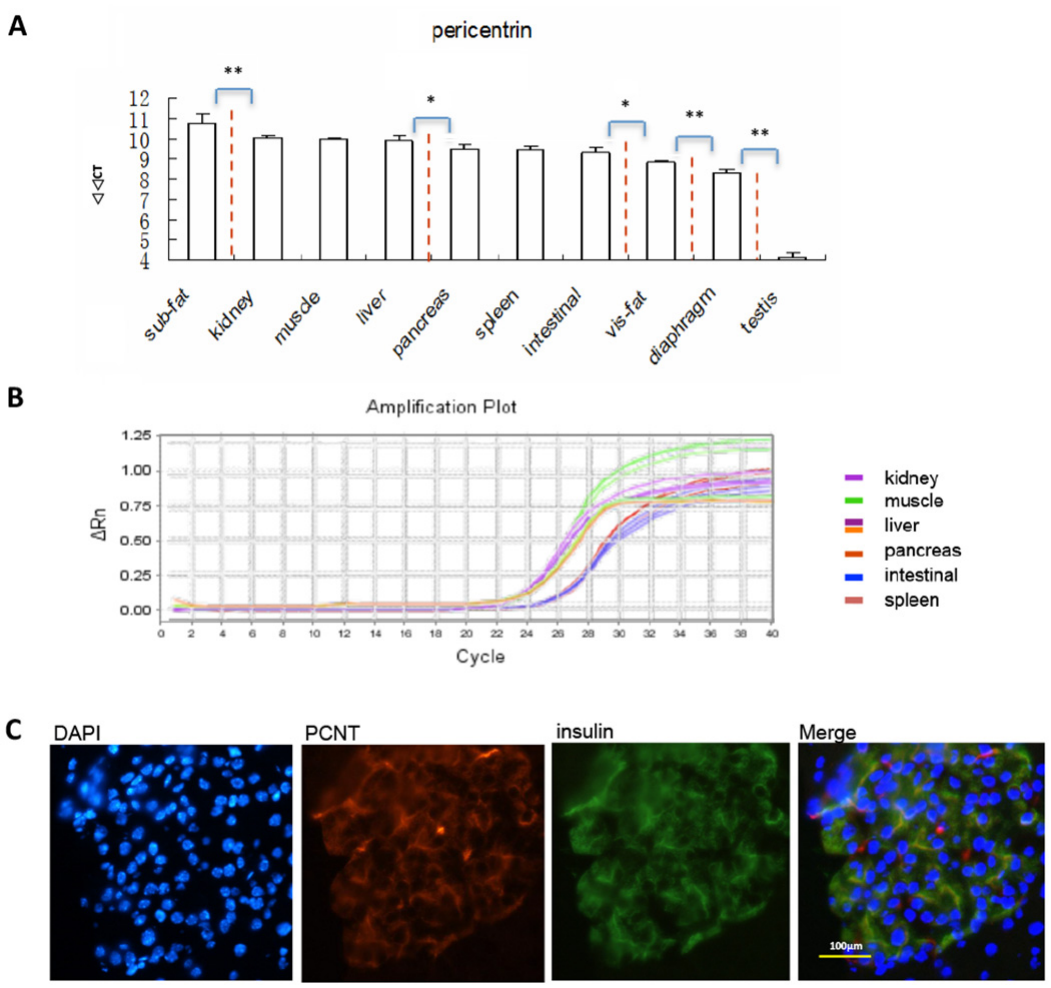
PCNT expression in tissues from various mouse organs. **(A)** A histogram showing RT-PCR results for PCNT expression in different mouse tissues. Tissue samples were divided into 6 groups according to their PCNT expression: Sub fat tissue had the highest expression of PCNT (group 1); kidney, muscle, liver had the second highest PCNT expression (group 2); PCNT expressions in pancreas, spleen, intestinal had the third highest expression (group 3); visceral fat, diaphragm, testis were the fourth, fifth and sixth highest, respectively (groups 4, 5, and 6; grouped by dash line). PCNT expression levels in different groups showed statistically significant differences, but were similar in replicates from the same group. **(B)** Amplification plot showing PCNT expression in different tissues from groups 2 and 3. Tissue samples were tested in triplicate. **(C)** Detection of PCNT expression by IF imaging in islets. PCNT was not only localized to centrosomes, but was also highly expressed throughout the cytoplasm. Insulin was stained using a FITC-conjugated secondary antibody (green), PCNT was stained using a Cy3-conjugated secondary antibody (red), and nuclei were stained with DAPI (blue).

Real-time PCR was used to compare differences in PCNT expression (Fig 1A and 1B). We found that PCNT was highly expressed in metabolic-regulating tissues, such as kidney, liver, muscle, and intestinal tissues. We also studied PCNT expression in islets by immunocytochemistry and immunofluorescence to study subcellular localization patterns. PCNT was not only expressed at the centrosomes, but was also highly expressed throughout the cytoplasm (Fig 1C). Our results showed that the subcellular localization of insulin and PCNT overlapped completely.

### Decreased PCNT expression in beta cells of high-fat-induced insulin resistant mice

**Fig 2:**
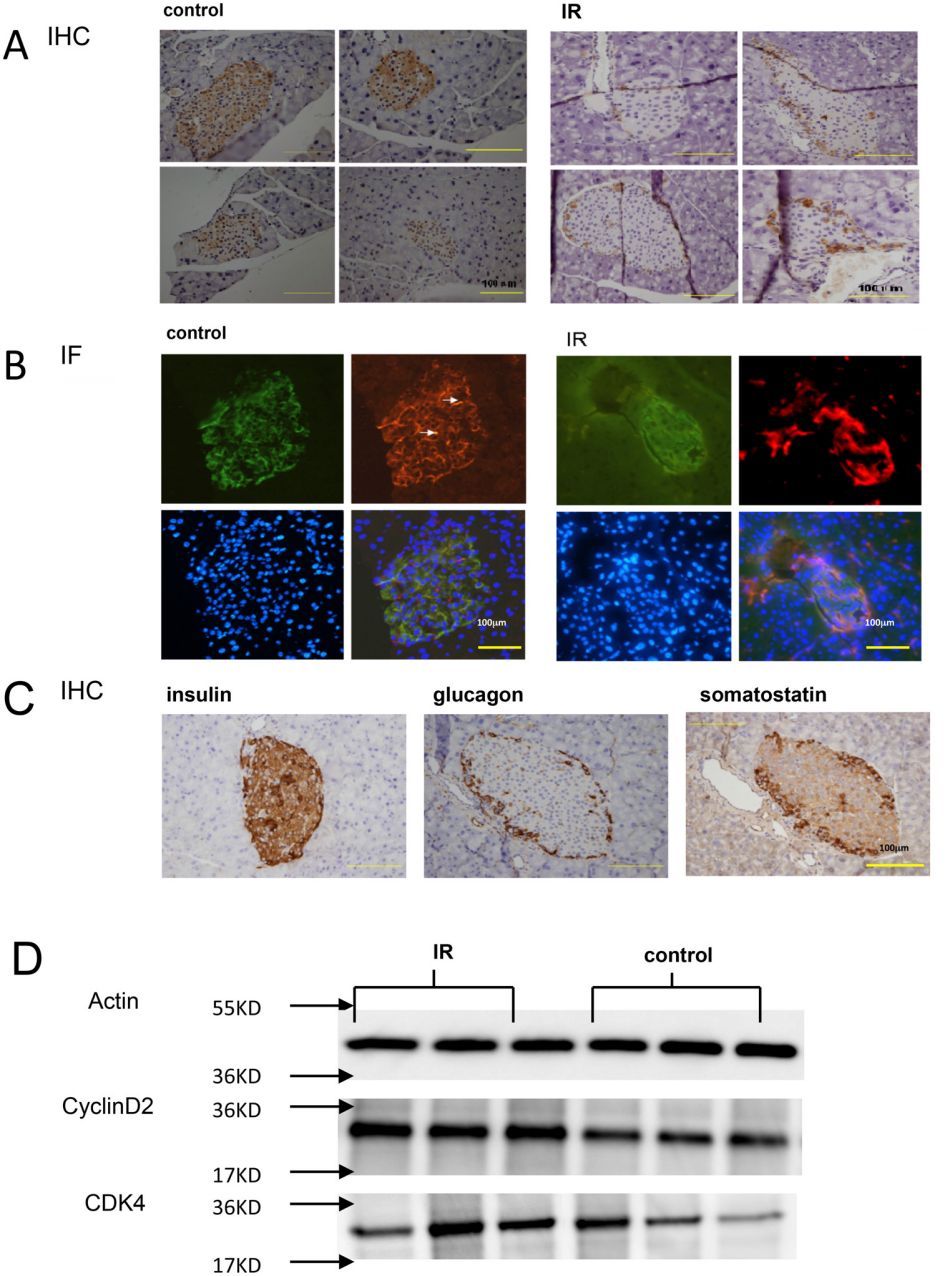
Comparison of PCNT expression in islets of IR and control mice. **(A)** IHC and **(B)** IF analysis of PCNT expression in the islets of IR and control mice. PCNT expression was significantly lower in the central region of islets in IR mice compared to those in the controls. **(C)** IHC staining for insulin, glycogen, and somatostatin expression in mouse islet cells. Beta cells showing robust insulin expression were centrally located in the islets and represented the majority of islet cells. Alpha cells expressed glucagon on the plasma membrane, and delta cells expressed cytoplasmic somatostatin and surrounded the islets.

Insulin resistant models in C57BL/6 mice were developed by feeding mice a high-fat diet for 12 weeks. Glucose tolerance tests showed that plasma glucose levels and the area under the glucose level-time curves were significantly higher in the IR group than in the control group. At week 12, HOMA-IR values of mice in the IR group were significantly higher compared to those in the controls (1.39 ± 0.14 vs. 0.44 ± 0.01, *P* < 0.01). IHC and IF results showed that PCNT expression was significantly decreased in IR mouse pancreatic tissue, mainly in the central part of the islets (Fig 2A and 2B). Insulin, glycogen, and somatostatin were stained in the islets cells (Fig 2C). Beta cells showing robust insulin expression were centrally located. This revealed that PCNT expression in central mouse islets might locate in beta cells. Moreover, the reduction in PCNT expression in central islets was not related to beta cell number, because the traditional proliferation indices (CyclinD2 and CDK4) were increased in western blot (Fig 2D).

### Decreased PCNT expression is related to abnormal insulin secretion

**Fig 3:**
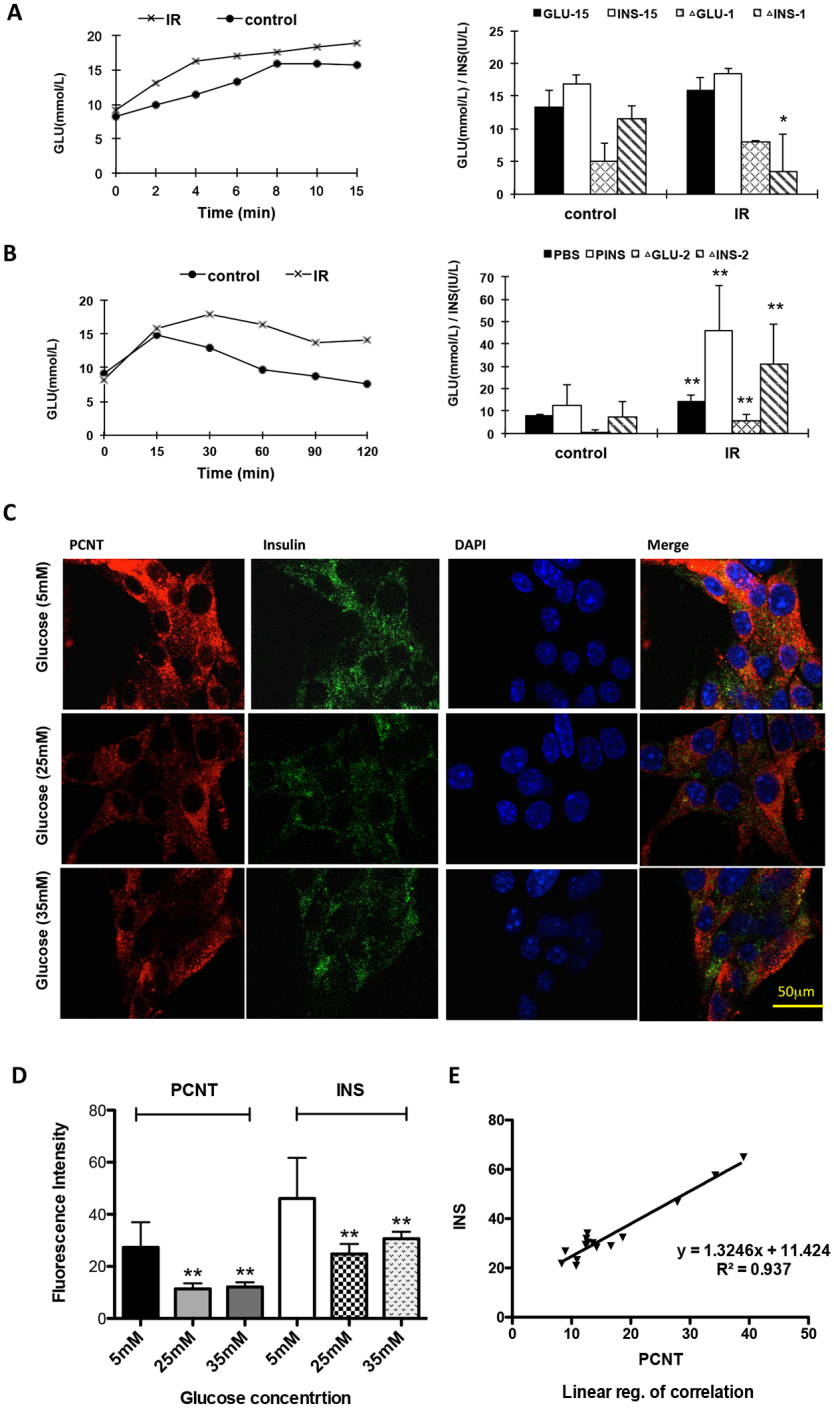
Insulin secretion in IR mice and PCNT and insulin expression following glucose stimulation in MIN6 cells. **(A)** First-phase IPGTT values in IR and control mouse groups. Dynamic change of glucose level at different time points was shown on line chart; final glucose level, final insulin level, the changes of glucose and insulin level in first-phase were shown on histogram cart. First-phase glucose levels in the IR mouse group were higher than those in the control group, while the increase of insulin levels were significantly lower in the IR group (*P* < 0.05). GLU: glucose level; GLU-15: glucose level at 15 min in IPGTT; INS-15: insulin level at 15 min in IPGTT; ΔGLU-1: change of first-phase glucose level; ΔINS-1: change of first-phase insulin level. **(B)** Second-phase OGTT values in IR and control mouse groups. Dynamic change of glucose level at different time points was shown on line chart; final glucose level, final insulin level, the changes of glucose and insulin level in second-phase were shown on histogram cart. Second-phase glucose and insulin levels in the IR group were higher than those observed in the control group (*P* < 0.01). PBS: glucose level at 2 h in OGTT; PINS: insulin level at 2 h in OGTT; ΔGLU-2: change of second-phase glucose level; ΔINS-2: change of second-phase insulin level. **(C)** Confocal microscopy imaging and **(D)** histogram showing PCNT and intracellular insulin staining in glucose stimulating MIN6 cells. Results are from quintuplicate experiments with duplicate wells. **(E)** The fluorescence change of PCNT and insulin in glucose stimulating MIN6 cells had linear relationships. Each plot represents one experiment with duplicate wells.

**Fig 4:**
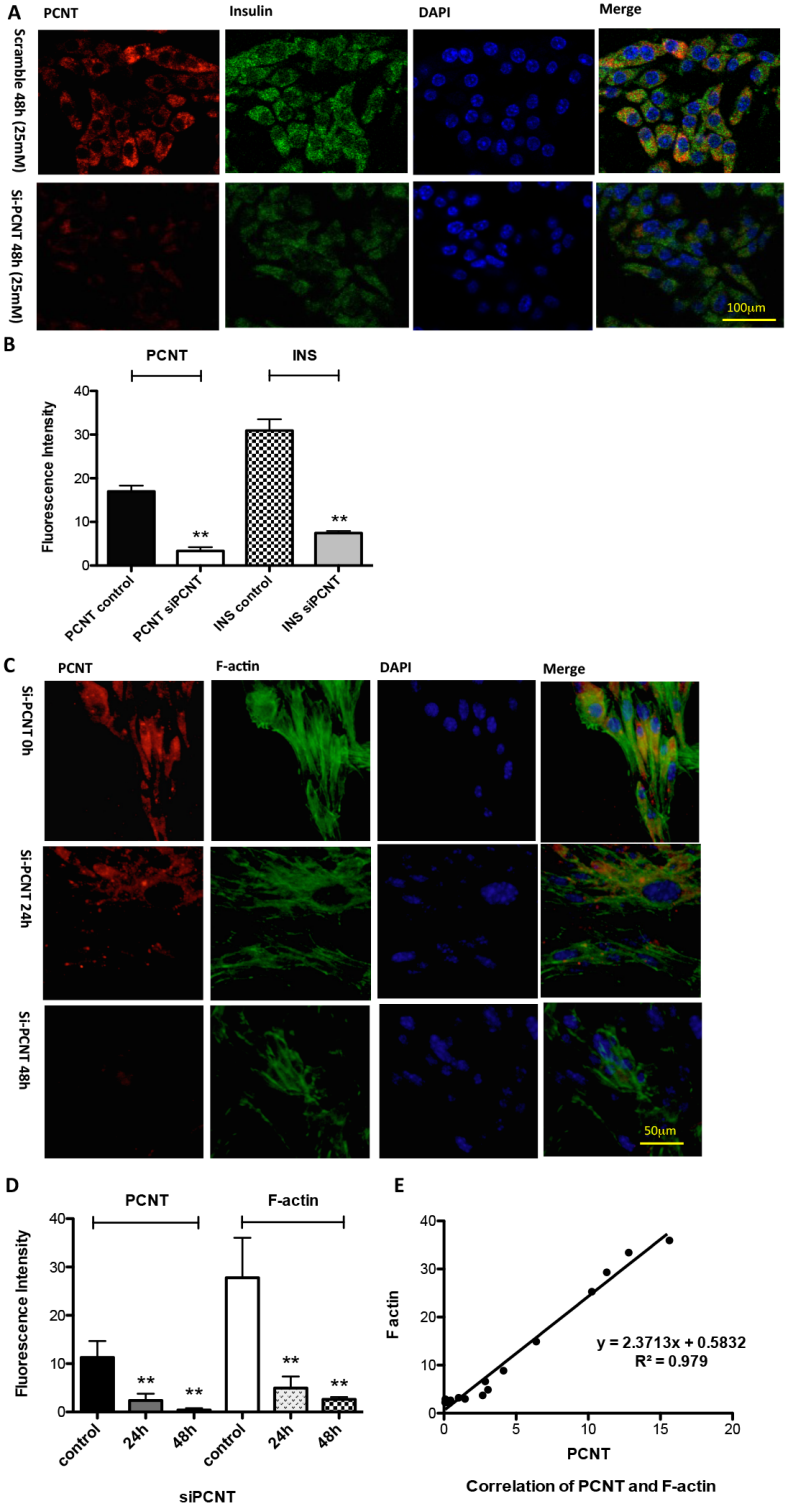
Change in F-actin, PCNT, and insulin expression following PCNT silencing. **(A)** Confocal microscopy imaging and **(B)** histogram show that intracellular insulin levels decreased significantly in MIN6 cells transfected with PCNT siRNA, compared with control cells transfected with a scrambled siRNA. **(C)** Confocal microscopy imaging and **(D)** histogram show florescence staining changes of F-actin in MIN6 cells after being transfected with a PCNT siRNA for 0 h, 24 h and 48 h. Results are from quintuplicate experiments with duplicate wells. **(E)** Linear relationship of F-actin and PCNT staining during 48 h of PCNT siRNA interference. Each plot represents one experiment with duplicate wells.

Intraperitoneal glucose bolus administration is a standard beta cell stimulus of first-phase insulin secretion. OGTT is a standard way to test the glucose tolerance mainly reflecting the function of the second phase. Compared with control mice, insulin secretion in IR mice was relatively lower in the first phase (lower ΔINS-1, Fig 3A), while it was significantly elevated in the second phase (higher ΔINS-2, Fig 3B). To test the possible effect of PCNT on insulin release, corresponding *in vitro* experiments were designed using MIN6 cells. In MIN6 cells exposed to glucose stimulation, PCNT and insulin expression levels decreased significantly from the 5 mM group to the 25 mM and 35 mM groups. The 25 mM group seemed to have the lowest PCNT and INS levels of the 3 groups. There was no significant difference between the 25 mM and 35 mM groups (Fig 3C and 3D). Linear regression revealed that there was a linear relationship between the fluorescence changes of PCNT and insulin in MIN6 cells under glucose stimulation (Fig 3E). The RNA interference experiment showed that an obvious decrease in intracellular insulin levels was observed in MIN6 cells transfected with PCNT siRNA compared with MIN6 cells transfected with a scrambled siRNA (Fig 4A and 4B).

### F-actin is related to PCNT in the regulation of insulin release

**Fig 5:**
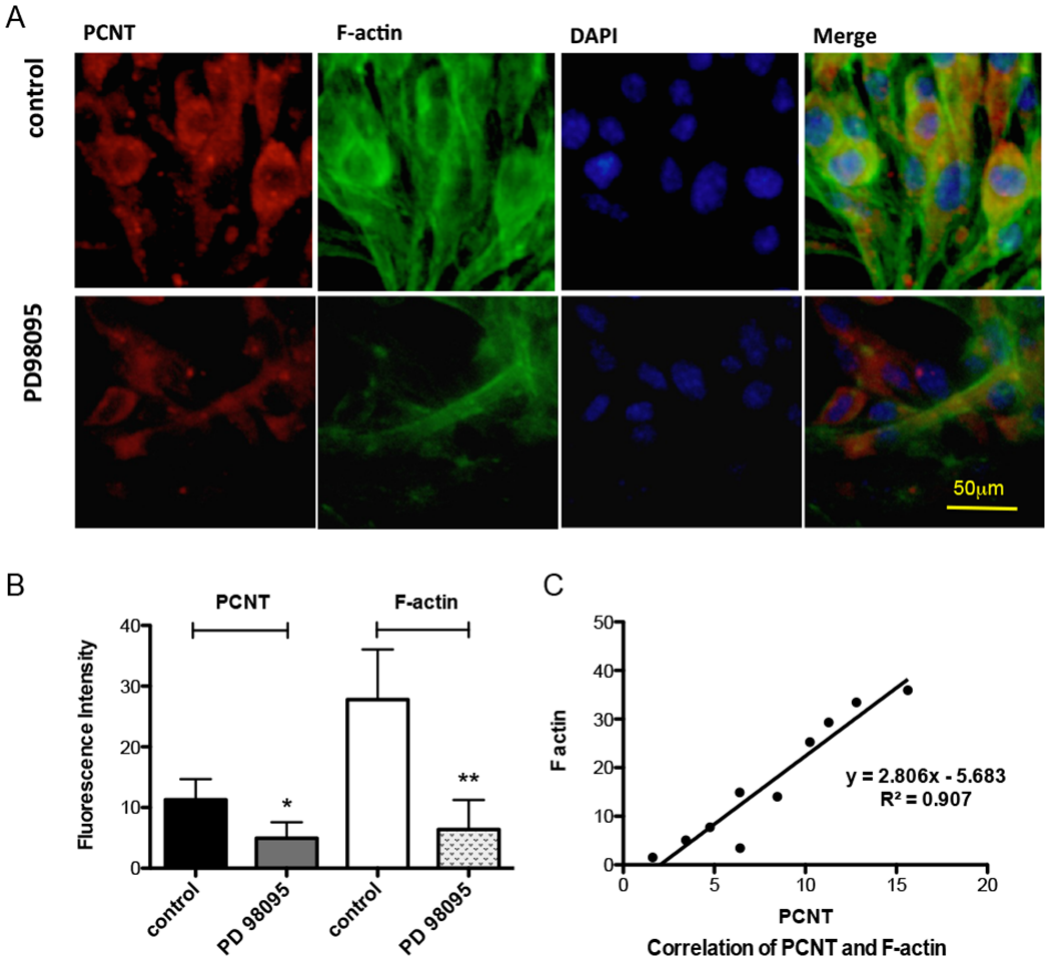
Relationship between PCNT and F-actin expression following exposure to ERK inhibitor (PD98095). **(A)** Confocal microscopy imaging and **(B)** histogram show that F-actin and PCNT fluorescence staining decreased significantly when MIN6 cells were exposed to the ERK inhibitor PD98085. Results are from quintuplicate experiments with duplicate wells. **(C)** F-actin and PCNT staining showed a linear relationship following exposure to the ERK inhibitor PD98085. Each plot represents one experiment with duplicate wells.

Gene chip results (Table 2) showed that F-actin was differentially expressed in pancreases of IR and control mice. F-actin expression was detected in MIN6 cells by IF. In MIN6 cells transfected with PCNT siRNA, the intracellular expression of F-actin was significantly decreased from 0 h, 24 h, to 48 h (Fig 4C and 4D). The fluorescence change of PCNT and F-actin had a linear relationship (Fig 4E). ERK was believed to be the upstream of F-actin. When the ERK inhibitor PD98095 was added to MIN6 cells, PCNT expression and F-actin were sharply decreased (Fig 5A and 5B). The relationship between the fluorescence of PCNT and F-actin in the control group and the PD98095 group remained linear (Fig 5C).

**Table 2:** Differentially expressed genes in IR and control mice, identified in the gene chip study.

Sequence number	Full name of the proteins	Ratio of IR/control
1367581_A_AT	secreted phosphoprotein 1	2.02
1367614_AT	annexin A1	2.04
1368271_A_AT	fatty acid binding proteins 4	3.05
1368397_AT	uridine diphosphate glucose	2.16
1370892	complement 4–2	2.02
1372190_AT	Aquaporin 4	2.14
1386901_AT	cluster of differentiation 36	2.27
1387683_AT	cluster of differentiation 36	2.14
1387995_A_AT	Interferon-induced transmembrane protein 3	2.05
1388583_AT	C-X-C motif chemokine 12	2.62
1377626_AT	calcium-activated chloride channels	2.39
1379582_A_AT	Cyclin A2	3.36
1379818_AT	clusterin	2.44
1381993_AT	chloride intracellular channel 2	2.11
1386695_AT	aryl-hydrocarbon receptor repressor	3.58
1394200_AT	hot shock proteins	2.29
1394220_AT	Cyclin D2	2.34
1367917_AT	F-actin	-2.03
1367811_AT	3-Phosphoglycerate dehydrogenase	-2.02
1368709_AT	fucosyltransferase 1	-2.37

## Discussion

In this study, PCNT was highly expressed in tissues and organs that are closely related with insulin resistance, such as subcutaneous fat, skeletal muscle, liver, and pancreas. Further IF staining revealed that PCNT is enriched only in the cytoplasm of islets. To our knowledge, this is the first time that PCNT expression has been shown to be significantly lower in the central region of IR mouse islets compared to that in control mice, where it is primarily composed of beta cells. The cytoplasmic expression of PCNT did not have a clear relationship with cellular proliferation in islets. For this reason, PCNT may serve functions other than cell division and cell cycle progression, which are the main functions of PCNT reported previously in cell types other than islets.

It is widely accepted that insulin is secreted in a biphasic manner through different mechanisms [18]. IR mice present an insufficient insulin release increase in the first phase and an abnormally high insulin level in second phase. Interestingly, we confirmed that the decreased PCNT was closely related to abnormal insulin release in glucose-stimulating MIN6 cells. Cytoplasm insulin fluorescence decreased sharply in high glucose (25 mM and 35 mM) stimulating MIN6 cells compared with low glucose (5 mM) cultured MIN6 cells. Cytoplasm fluorescence of PCNT changed in accordance with that of insulin, for which there was a linear relationship. Furthermore, when PCNT expression was down-regulated by RNA interference, a decrease in intracellular insulin vesicles occurred. These results suggest that the significant decrease in PCNT expression in beta cells was closely related with abnormal insulin release, which might contribute to the abnormal insulin secretion in insulin-resistant mice.

Next, we focused on the mechanism underlying beta cell insulin secretion. Gene chips showed that the most differentially expressed genes between IR and control mice were related to metabolism, proliferation, apoptosis, inflammation, and cytoskeletal functions. In our studies, down-regulation of PCNT siRNA significantly decreased F-actin expression in immunofluorescence. There was a linear relationship between the decreased fluorescence of PCNT and F-actin during RNA interference. ERK was thought to be upstream in regulating F-actin polymerization conditions; it was also thought that ERK could be activated by glucose. Interestingly, in our study, the ERK inhibitor PD98059 significantly decreased the immunofluorescence detection of F-actin and PCNT in MIN6 cells. The decrease in PCNT was linear in relation to that of F-actin. Therefore, we believe that F-actin depolymerization may be regulated by PCNT. Landmark research by Orci et al. [19] showed electron micrographs of islet beta cells depicting F-actin in the form of a meshwork barrier just beneath the plasma membrane, which might have the effect of preventing the release of insulin. Jurczyk et al. [20] reported that a possible function of PCNT was to mediate transport vesicle docking in islet beta cells. Thus the change of F-actin expression, which is related to the decrease in PCNT, might explain the abnormal insulin secretion observed in IR mice.

In conclusion, abnormal insulin secretion observed both *in vivo* and *in vitro* was associated with decreased PCNT expression, and F-actin was found to be the target of PCNT regulation. The decrease of PCNT expression in beta cells plays an important role in the abnormal insulin secretion in IR pathologic procedure. PCNT may be a novel target for modulating regulated protein secretion in disorders such as insulin resistance or diabetes. Research into the effect of PCNT on insulin release may give a new way to prevent and control diabetes in a clinical setting.

## References

[1] Shi Y, Hu FB (2014) The global implications of diabetes and cancer. Lancet 383: 1947–1948. 10.1016/S0140-6736(14)60886-2 24910221

[2] Vos T, Flaxman AD, Naghavi M, Lozano R, Michaud C, Ezzati M, et al (2012) Years lived with disability (YLDs) for 1160 sequelae of 289 diseases and injuries 1990–2010: a systematic analysis for the Global Burden of Disease Study 2010. Lancet 380: 2163–2196. 10.1016/S0140-6736(12)61729-2 23245607 PMC6350784

[3] Wild S, Roglic G, Green A, Sicree R, King H (2004) Global prevalence of diabetes: estimates for the year 2000 and projections for 2030. Diabetes Care 27: 1047–1053. 15111519 10.2337/diacare.27.5.1047

[4] Mathers CD, Loncar D (2006) Projections of global mortality and burden of disease from 2002 to 2030. PLoS Med 3: e442 17132052 10.1371/journal.pmed.0030442 PMC1664601

[5] Henquin JC, Boitard C, Efendic S, Ferrannini E, Steiner DF, Cerasi E (2002) Insulin secretion: movement at all levels. Diabetes 51 Suppl 1: S1–2. 11815449 10.2337/diabetes.51.2007.s1

[6] Ma D, Shield JP, Dean W, Leclerc I, Knauf C, Burcelin R Ré, et al (2004) Impaired glucose homeostasis in transgenic mice expressing the human transient neonatal diabetes mellitus locus, TNDM. J Clin Invest 114: 339–348. 15286800 10.1172/JCI19876 PMC484972

[7] Olofsson CS, Gopel SO, Barg S, Galvanovskis J, Ma X, Salehi A, et al (2002) Fast insulin secretion reflects exocytosis of docked granules in mouse pancreatic B-cells. Pflugers Arch 444: 43–51. 11976915 10.1007/s00424-002-0781-5

[8] Rorsman P, Eliasson L, Renstrom E, Gromada J, Barg S, et al (2000) The Cell Physiology of Biphasic Insulin Secretion. News Physiol Sci 15: 72–77. 11390882 10.1152/physiologyonline.2000.15.2.72

[9] Doxsey SJ, Stein P, Evans L, Calarco PD, Kirschner M (1994) Pericentrin, a highly conserved centrosome protein involved in microtubule organization. Cell 76: 639–650. 8124707 10.1016/0092-8674(94)90504-5

[10] Jurczyk A, Gromley A, Redick S, San Agustin J, Witman G, Pazour GJ, et al (2004) Pericentrin forms a complex with intraflagellar transport proteins and polycystin-2 and is required for primary cilia assembly. J Cell Biol 166: 637–643. 15337773 10.1083/jcb.200405023 PMC2172416

[11] Martinez-Campos M, Basto R, Baker J, Kernan M, Raff JW (2004) The Drosophila pericentrin-like protein is essential for cilia/flagella function, but appears to be dispensable for mitosis. J Cell Biol 165: 673–683. 15184400 10.1083/jcb.200402130 PMC2172389

[12] Miyoshi K, Asanuma M, Miyazaki I, Matsuzaki S, Tohyama M, Oqawa N (2006) Characterization of pericentrin isoforms in vivo. Biochem Biophys Res Commun 351: 745–749. 17084386 10.1016/j.bbrc.2006.10.101

[13] Miyoshi K, Onishi K, Asanuma M, Miyazaki I, Diaz-Corrales FJ, Oqawa N (2006) Embryonic expression of pericentrin suggests universal roles in ciliogenesis. Dev Genes Evol 216: 537–542. 16534625 10.1007/s00427-006-0065-8

[14] Delaval B, Doxsey SJ (2010) Pericentrin in cellular function and disease. J Cell Biol 188: 181–190. 10.1083/jcb.200908114 19951897 PMC2812529

[15] Miyoshi K, Kasahara K, Miyazaki I, Shimizu S, Taniguchi M, Matsuzaki S, et al (2009) Pericentrin, a centrosomal protein related to microcephalic primordial dwarfism, is required for olfactory cilia assembly in mice. FASEB J 23: 3289–3297. 10.1096/fj.08-124420 19470799

[16] Muhlhans J, Brandstatter JH, Giessl A (2011) The centrosomal protein pericentrin identified at the basal body complex of the connecting cilium in mouse photoreceptors. PLOS ONE 6: e26496 10.1371/journal.pone.0026496 22031837 PMC3198765

[17] Ishihara H, Asano T, Tsukuda K, Katagiri H, Inukai K, Anai M, et al (1993) Pancreatic beta cell line MIN6 exhibits characteristics of glucose metabolism and glucose-stimulated insulin secretion similar to those of normal islets. Diabetologia 36: 1139–1145. 8270128 10.1007/BF00401058

[18] Wang Z, Thurmond DC (2009) Mechanisms of biphasic insulin-granule exocytosis—roles of the cytoskeleton, small GTPases and SNARE proteins. J Cell Sci 122: 893–903. 10.1242/jcs.034355 19295123 PMC2720925

[19] Orci L, Gabbay KH, Malaisse WJ (1972) Pancreatic beta-cell web: its possible role in insulin secretion. Science 175: 1128–1130. 4551150 10.1126/science.175.4026.1128

[20] Jurczyk A, Pino SC, O'Sullivan-Murphy B, Addorio M, Lidstone EA, Diiorio P, et al (2010) A novel role for the centrosomal protein, pericentrin, in regulation of insulin secretory vesicle docking in mouse pancreatic beta-cells. PLOS ONE 5: e11812 10.1371/journal.pone.0011812 20676397 PMC2910730

